# Identification of influenza A nucleoprotein body domain residues essential for viral RNA expression expose antiviral target

**DOI:** 10.1186/s12985-017-0694-8

**Published:** 2017-02-07

**Authors:** Alicia M. Davis, Jose Ramirez, Laura L. Newcomb

**Affiliations:** 1 Department of Biology, California State University San Bernardino, San Bernardino, CA USA; 2 Present Address: Irell & Manella Graduate School of Biological Sciences, City of Hope, Duarte, CA USA; 3 Present Address: Tufts University School of Medicine, Boston, MA USA

**Keywords:** Influenza, Virus, RNA, Nucleoprotein

## Abstract

**Background:**

Influenza A virus is controlled with yearly vaccination while emerging global pandemics are kept at bay with antiviral medications. Unfortunately, influenza A viruses have emerged resistance to approved influenza antivirals. Accordingly, there is an urgent need for novel antivirals to combat emerging influenza A viruses resistant to current treatments. Conserved viral proteins are ideal targets because conserved protein domains are present in most, if not all, influenza subtypes, and are presumed less prone to evolve viable resistant versions. The threat of an antiviral resistant influenza pandemic justifies our study to identify and characterize antiviral targets within influenza proteins that are highly conserved. Influenza A nucleoprotein (NP) is highly conserved and plays essential roles throughout the viral lifecycle, including viral RNA synthesis.

**Methods:**

Using NP crystal structure, we targeted accessible amino acids for substitution. To characterize the NP proteins, reconstituted viral ribonucleoproteins (vRNPs) were expressed in 293 T cells, RNA was isolated, and reverse transcription – quantitative PCR (RT-qPCR) was employed to assess viral RNA expressed from reconstituted vRNPs. Location was confirmed using cellular fractionation and western blot, along with observation of NP-GFP fusion proteins. Nucleic acid binding, oligomerization, and vRNP formation, were each assessed with native gel electrophoresis.

**Results:**

Here we report characterization of an accessible and conserved five amino acid region within the NP body domain that plays a redundant but essential role in viral RNA synthesis. Our data demonstrate substitutions in this domain did not alter NP localization, oligomerization, or ability to bind nucleic acids, yet resulted in a defect in viral RNA expression. To define this region further, single and double amino acid substitutions were constructed and investigated. All NP single substitutions were functional, suggesting redundancy, yet different combinations of two amino acid substitutions resulted in a significant defect in RNA expression, confirming these accessible amino acids in the NP body domain play an important role in viral RNA synthesis.

**Conclusions:**

The identified conserved and accessible NP body domain represents a viable antiviral target to counter influenza replication and this research will contribute to the well-informed design of novel therapies to combat emerging influenza viruses.

## Background

Influenza A viruses cause seasonal respiratory infections that lead to many hospitalizations and deaths each year. The Influenza A virus genome is comprised of eight negative sense single stranded RNA (vRNA) segments. Humans, avians, and swine are all susceptible to influenza A virus. Cases of direct avian to human transmission are rare [1] because humans and avians are susceptible to specific subtypes of influenza A virus [2]. Pigs however, are susceptible to infection with human, avian, and swine influenza subtypes, allowing for the mixing of genomic segments between various subtypes of the virus and the potential for a new pandemic influenza A subtype to emerge. Genome reassortment through segment mixing can yield new Influenza A subtypes of varying transmissibility and pathogenicity. Reassortant viruses have the potential to cause human pandemics, as seen in 1918, 1957, 1968, and most recently 2009 [3].

Annual vaccines are used to help protect against several subtypes of the Influenza A virus and two subtypes of Influenza B. However, because vaccine production takes months, circulating viruses can mutate and reassort while vaccine production is ongoing, resulting in decreased vaccine effectiveness. Indeed, both mutation and reassortment of influenza genes reduce efficacy of yearly vaccines; resulting in at best 23% vaccine effectiveness according to the CDC [4]. It was recently reported that the nasal spray vaccine known as FluMist was ineffective and did not offer protection from the virus, with the CDC’s Advisory Committee on Immunization Practices (ACIP) voting down live attenuated influenza vaccine (LAIV) for use during the 2016–2017 season [5]. Similarly, due to the production time required to generate vaccines, they are not an option to protect against newly emerging subtypes of influenza virus, as seen in 2009 with the novel H1N1 pandemic. Once infection has occurred, antiviral drugs are taken to aid in recovery and antiviral drugs were essential to slow the spread of the 2009 pandemic [6]. Current antivirals fall under two categories, neuraminidase inhibitors (oseltamivir and zanamivir) and M2 ion channel blockers (amantadine and rimantadine). The drugs targeting the M2 ion channel are no longer efficacious due to resistance that has developed within the circulating strains of influenza. The widely publicized antiviral drug Tamiflu (oseltamivir) is still in use, although gene segments encoding resistance are in circulation as there have been a small number of viral subtypes found to be resistant to treatment with Tamiflu [7]. Other evidence demonstrates resistance to oseltamivir can be selected for during treatment [8–11]. Continued use of Tamiflu and other neuraminidase inhibitors will select for emergence of resistant strains and it is possible neuraminidase inhibitors will lose effectiveness and no longer be of use, as observed with the M2 ion channel inhibitors. With the potential of a dangerous influenza pandemic arising, new antiviral drugs targeting conserved regions of the virus are urgently needed [12].

**Fig. 1:**
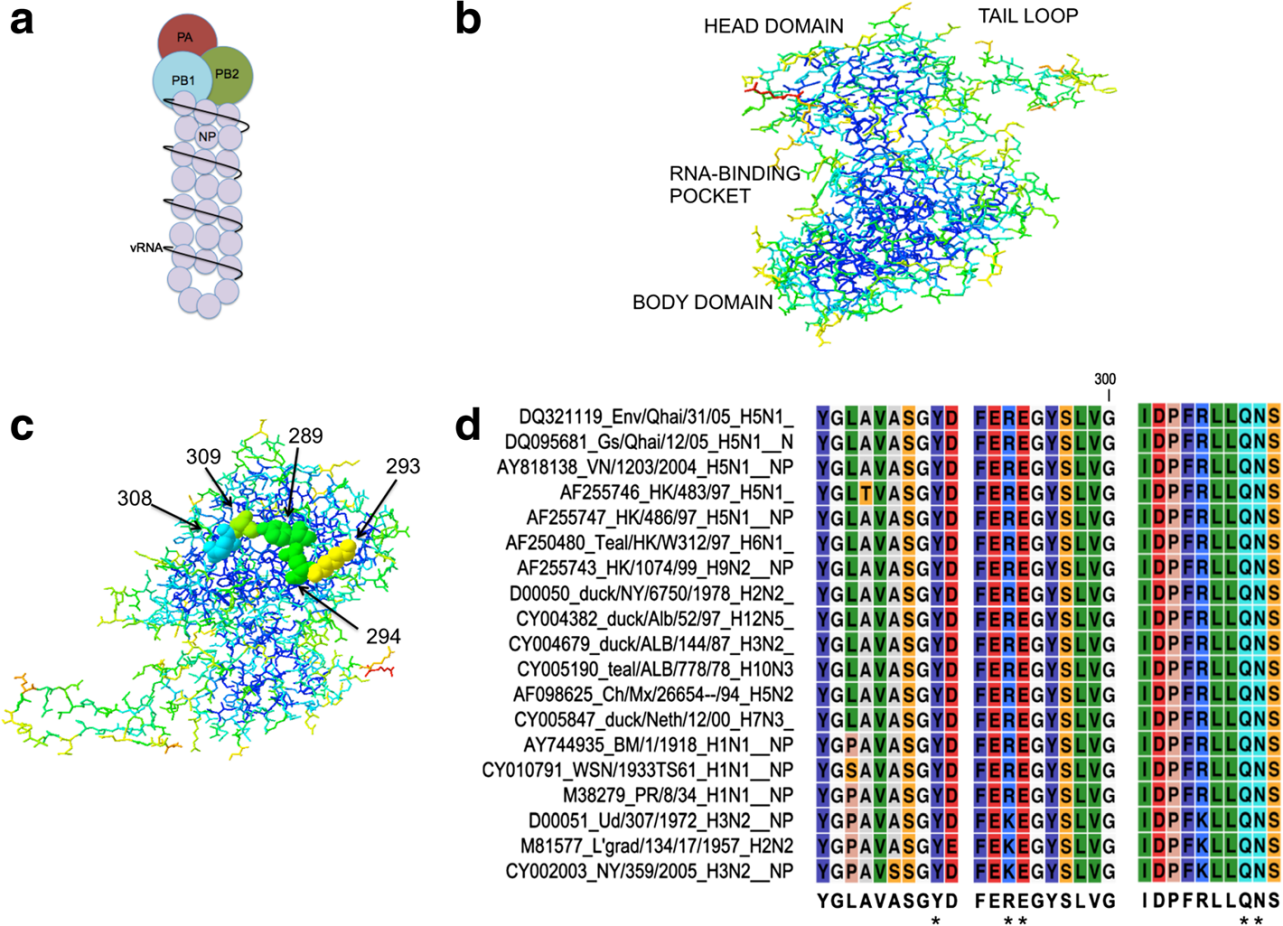
Components of the Viral Ribonucleoprotein and Nucleoprotein. **a** Graphical illustration of the viral Ribonucleoprotein (vRNP) complex, comprised of the viral polymerase subunits, PB1, PB2, and PA, bound to both the 5′ and 3′ ends of the viral RNA segment, and multiple copies of nucleoprotein (NP). **b** Domains of Nucleoprotein monomer crystal structure [32] using Deep View-Swiss-PdbViewer 4.0, with head domain, body domain, RNA pocket, and tail loop regions labeled. **c**. Nucleoprotein body domain substitutions of NPbd3 using Deep View-Swiss-PdbViewer 4.0 to analyze the accessible residues in the body domain of the NP monomer crystal structure [32]. In both **b** and **c**, residue color represents accessibility within the NP monomer as determined by the Deep View-Swiss-PdbViewer 4.0 “color” tool, with greatest to least accessible as follows: red, orange, yellow, green, light blue, and dark blue. Residues 289, 293, 294, 308, and 309 mutated in NPbd3 are highlighted in C. **d** Nucleoprotein (NP) sequence alignment from select influenza subtypes using CLS freeware and amino acids 281-310. Colors correlate with amino acid property. Asterisks indicate NPbd3 mutant

The Influenza A virus utilizes eight genomic segments to encode at least ten mRNAs through alternate splicing [13] and yield greater than twelve proteins through alternate translation [14–17]. There are two viral surface proteins, HA and NA, for which the influenza subtypes are named. The RNA dependent RNA polymerase (RdRP) complex is comprised of three proteins: PB1, PB2, and PA. Nucleoprotein (NP) binds the single stranded genome segments and interacts with the RdRP to form the viral ribonucleoprotein (vRNP), responsible for RNA synthesis (Fig. 1a). The remaining two segments are M and NS, which are alternately spliced to form M1, M2, NS1, and NS2 (NEP) proteins.

Upon infection the eight vRNPs are transported to the nucleus to transcribe and replicate the vRNA. The PB2 subunit contains the active site that binds to the host pre-mRNA [18] while the PA subunit of the polymerase complex cleaves a cellular capped-mRNA primer from host mRNA to initiate transcription [19–21]. The PB1 subunit uses this short, capped host RNA to carry out polymerization of the viral mRNA transcript. Termination of transcription occurs when the polymerase reaches a repetitive sequence of U residues and stutters, which produces a poly(A) tail [22]. Viral replication off the vRNA template results a full-length complementary (cRNA) intermediate, which is then replicated to yield progeny vRNA.

NP is more than just a structural component of the vRNP as NP interacts with both viral [23, 24] and host factors [25–29] to regulate viral RNA expression. Fifty-nine percent of NP residues are highly conserved among influenza A isolates [30] making NP interactions compelling antiviral targets [12]. NP is comprised of two main regions referred to as the head domain and the body domain (Fig. 1b). In between the head and body domain is a positively charged RNA binding groove. The negatively charged phosphate backbone of viral vRNA and cRNA forms ionic bonds with the arginine-rich groove and contributes to the overall shape of the vRNP [31]. Opposite the RNA binding groove is the portion of the protein referred to as the tail loop. NP monomers undergo oligomerization through this tail loop consisting of a salt bridge between residues 339 and 416 on different NP monomers [32–34]. NP oligomerization is strengthened by RNA binding and is essential for the formation of functional vRNPs [34]. NP also directly interacts with components of the RdRP including PB1 and PB2 [32, 35]. Several groups have identified the body domain of NP as a site of interaction with PB2 [32, 35, 36].

Here we characterize mutations within 5 amino acids of NP that comprise an accessible region of the NP body domain, as determined by NP crystal structure [32]. This region was selected for mutagenesis to target interaction between NP and RdRP [35]. The amino acids that were substituted were chosen based on relative sequence conservation and accessibility in regards to the surface of the protein determined by examining the cryo-electron microscopy (cryo-EM) structure of mini-vRNPs [37] and monomer NP crystal structure [32] analyzed for side chain accessibility using Deep View-Swiss-PdbViewer 4.0. Five glycine substitutions were sufficient to completely shut down NP function in viral RNA synthesis. Despite these findings, single glycine substitutions within this region were as functional as wild type NP. Double mutants in this region exhibited partial activity, indicating that this surface is likely comprised of several amino acids important for interaction in functional vRNPs. Our findings highlight this conserved NP domain as an important interaction surface essential for viral RNA synthesis and support further investigation of antiviral drugs that target this region of NP.

## Methods

### Cells

293 T Human embryonic kidney cells were grown in a water-jacketed incubator with 5% CO_2_ output at 37 °C. The cell line was purchased from ATCC.

### Plasmids

pcDNA NP-FLAG, PA, PB1, PB2 plasmids were used to encode Influenza A/Udorn/307/72 (H3N2) mRNA to drive expression of the viral proteins required for vRNP formation. NP is fused to a C-terminal 1X FLAG epitope tag and results in functional vRNPs and cRNPs [38].

pHH21 GFP-M vRNA and pHH21 M cRNA plasmids were used to express either GFP-M vRNA (- sense) or M cRNA (+ sense) to complete vRNP or cRNP expression. The expressed M cRNA or GFP-M vRNA must be processed by the viral polymerase ribonucleoprotein to be expressed as mRNA for translation to protein.

For this study, sixteen plasmids to express the different NP glycine substitution and two plasmids to express NP GFP fusion proteins were constructed by two-step PCR. Briefly, in the first step, two individual PCR reactions generated two fragments with complementary ends. A second PCR reaction combined the two fragments to generate the entire portion of DNA and include restriction enzyme sites for digestion and ligation into the pcDNA3 vector. Constructed plasmids were sequenced confirmed by Retrogen.

### DNA transfection

Plasmid mixtures to express reconstituted vRNPs and cRNPs were prepared including pcDNA PB1, pcDNA PB2, pcDNA PA, and pcDNA WT NP-FLAG, pcDNA Vector (no NP), or NP mutant along with pHH21 GFP-MvRNA or McRNA to express vRNA or cRNA respectively. RNAs must be processed by the viral polymerase ribonucleoprotein to be expressed as mRNA for translation to protein. Additional experiments transfected single plasmids at suggested concentration per culture dish. In all cases, DNA was transfected with Trans-IT reagent (DNA to reagent ratio of 1:3) as per manufacturer’s directions. Cells were incubated in a tissue culture incubator for 48 h using either 6-well dishes or 100 mm dishes as appropriate for experiment.

### GFP visualization representing reconstituted vRNP and cRNP activity

Cells were observed for GFP-M expression 48 h post-transfection. WT NP served as positive control while no NP is negative control. GFP was visualized with a Nikon Eclipse TS100 (Nikon Intensilight C-HGFI for fluorescence) inverted microscope and images captured with the Nikon DS-Qi1Mc camera with NS Elements software.

### Cell collection

Forty-eight hours post-transfection cells were collected and pelleted by centrifugation. Proteins were isolated through either total protein isolation or cellular fractionation.

### Total protein isolation

Cell pellets were resuspended in RIPA Lysis Buffer (25 mM HCl pH 7.6, 150 mM NaCl, 1% deoxycholate, 0.1% SDS) containing protease inhibitors and lysed using a Fisher Scientific Sonic Dismembrator for 10 pulses at 30%, output 3–4. Soluble protein extract was clarified with high-speed centrifugation to pellet debris. Proteins were denatured using 1X SDS protein loading dye and heat at 95 °C for five minutes. Proteins were separated on a 10% sodium dodecyl sulfate polyacrylamide gel electrophoresis (SDS PAGE) and transferred to nitrocellulose.

### Cellular fractionation

Cells were fractionated using 0.2% NP-40 non-ionic detergent in RSB to break open cellular plasma membrane while keeping the nuclear membranes intact. Microscopy was used to confirm disrupted plasma membranes and intact nuclei. The nuclei were pelleted by centrifugation, resuspended in RSB, and sonicated for 30 pulses at 30% to bust the nuclear membrane. Both nuclear and cytoplasmic fractions were clarified by high-speed centrifugation.

### Western blot

All NP constructs encode a FLAG epitope tag at the C-terminus and were detected using anti-FLAG antibody (Agilent). Anti-tubulin antibody (Abcam) was used to confirm protein loading when evaluating total protein. Anti-Hsp90 antibody (Abcam) to detect Hsp90, a protein localized in the cytoplasm, serves as confirmation of cellular fractionation. Pierce ECL reagents (Thermo Scientific) were used to develop blots and images were captured using a Chemidoc XRS imager (Bio-Rad) with Quantity One software.

### Immunopurification

The nuclear fraction of cells transfected with WT-NP, NPbd3, or No NP were incubated with Anti- FLAG M2 Affinity Gel agarose beads (Sigma-Aldrich), incubated overnight at 4 °C with rotation, washed five times in RSB (reticulocyte standard buffer: 10 mM Tris HCl pH 7.5, 10 mM KCl, 1.5 mM MgCl_2_) + 0.2% NP-40, and eluted for 1 h at room temperature with 150 ng/ul 3X FLAG peptide (APEXBIO).

### Electrophoretic mobility shift assay (EMSA)

Similar volume of immunopurified proteins were incubated with 20 picomoles of biotin labeled ssDNA for 20 min at room temperature and mixed with 2.5% glycerol for loading. The protein-DNA samples were run on an 8% TBE non-denaturing PAGE in 4 °C, followed by transfer to nitrocellulose and Western blotting. The membrane was probed with Streptavadin-HRP (1:3000) and Pierce ECL reagents were used to detect the biotin labeled ssDNA.

### Blue native polyacrylamide gel electrophoresis (BN-PAGE)

Total protein from WT-NP, NPbd3, or no NP transfected cells were run on a 6% 1D Blue Native PAGE while total protein from cells expressing reconstituted vRNPs was run on a gradient of 4–13% 1D Blue Native PAGE. Resolved complexes were transferred on to nitrocellulose and blots were probed with anti-FLAG to detect WT-NP and NPbd3.

### GFP fusion protein location

Plasmids expressing NP-GFP, NPbd3-GFP, or eGFP were transfected into 293 T cells grown on poly-L-Lysine cover slips. 48 h post transfection the cells were washed and fixed using a 1:1 methanol and acetone mixture. The coverslips were mounted onto glass slides using SouthernBiotech™ DAPI-Fluoromount-G™ Clear Mounting Media, which stains the cell nucleus blue. Slides were observed on a Nikon ECLIPSE TE2000-U fluorescent microscope and images were captured with an Andor Clara DR-3446 camera using NIS-Elements AR software.

### RNA isolation

Total RNA was isolated with Trizol (Invitrogen), following the manufacturer’s protocol. RNA concentration and purity of samples was determined at OD_260_ and OD_280_ using a NanoDrop ND1000 Nanospectrophotometer (Thermo Fischer Scientific). Integrity of rRNA was evaluated on a 1% bleach/1% agarose gel [39].

### RTqPCR

2.5 micrograms (μg) of RNA was DNase treated. 1 μg was reverse transcribed using Promega AMV reverse transcription system following the manufacturer’s protocol. 1 μg was used in the negative control reaction, lacking the AMV reverse transcriptase enzyme to control for DNA contamination. Oligo dT (mRNA) or vRNA specific primers (vRNA) were used in the RT reaction to produce cDNA. Real time quantitative PCR using an Applied Biosystems SYBR Select Master Mix was performed on cDNA with primers targeting the influenza M gene. qPCR reactions were carried out in triplicate using the AB Step One qPCR machine. Significance was evaluated through t-test by comparing WT NP with no NP or NPbd3 both reporting significance with *p*-values <0.02.

## Results

### Mutational analysis of nucleoprotein domains

Accessible regions of the RNA binding pocket, head domain, and body domain were examined by amino acid substitutions created through cloning. Accessibility was characterized using the Deep View Swiss PdbViewer “color” toolbar. The RNA binding pocket mutant, NPrbp1, encoded R152A while the head domain mutant, NPhd1 was altered at amino acids R213A and K214A. Five regions of the NP body domain were investigated through the following mutants: NPbd1 (E46A, K48A), NPbd2 (D101A), NPbd3 (Y289G, K293G, E294G, Q308G, N309G), NPbd4 (S482A), and NPbd5 (S467G, E469G, N470G, T471G, and N472G). Both Alanine and Glycine are used in substitution analysis to remove interacting side groups.

**Fig. 2:**
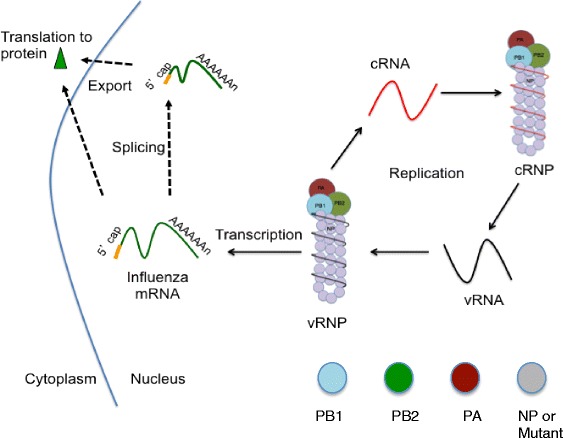
Reconstituted RNP Expression System. 293 T cells are transfected with plasmids to express mRNAs encoding the viral RdRP and NP or NP substitution mutants, and a specific vRNA or cRNA template. Cellular RNA polymerase II drives expression of viral mRNAs from pcDNA3 plasmid backbone while cellular RNA polymerase I drives expression of the cRNA or vRNA template from pHH21 plasmid backbone. vRNP function is assessed by gene expression from vRNA or cRNA templates

**Fig. 3:**
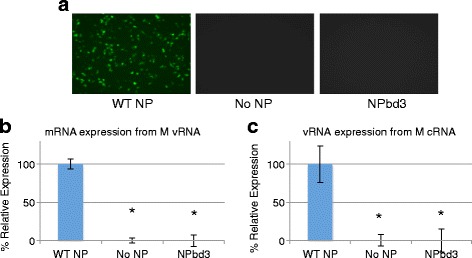
NPbd3 is defective for viral protein and RNA expression in reconstituted vRNPs. **a** Plasmids to express reconstituted vRNPs with GFP-M vRNA and either WT-NP, no NP, or NPbd3 were transfected into 293 T cells. 48 h post transfection cells were observed for GFP-M expression. WT NP represents the positive control while no NP is the negative control. GFP was visualized with a Nikon Eclipse TS100 (Nikon Intensilight C-HGFI for fluorescence) inverted microscope and images captured with the Nikon DS-Qi1Mc camera with NS Elements software. **b** and **c** RNA was purified from cells expressing reconstituted vRNPs (**b**) or cRNPs (**c**) as indicated. 1 μg was DNase treated and subject to reverse transcription with oligo dT (**b**) or vRNA specific primers (**c**) and quantitative PCR with M gene specific primers to calculate relative M RNA expression in each sample. Data are from triplicate trials; asterisks indicate *p* < 0.02

Activity of each NP mutant was tested using the reconstituted viral ribonucleoprotein (vRNP) expression system (Fig. 2). Plasmids to express mRNA encoding PB1, PB2, PA, and either NP, no NP, or NP mutant, were transfected into 293 T cells along with plasmid to express Green Fluorescent Protein (GFP) M vRNA, the negative sense vRNA segment of the reconstituted vRNP. GFP positive cells within this system represent viral gene expression. GFP expression was observed 48 h post transfection. All NP mutants expressed GFP at similar levels as wild type NP (WT-NP) except NPbd3, the third of five body domains mutants constructed (Table 1 and Fig. 3a). NPbd3 was investigated further.

**Table 1:** NP amino acid substitution analysis

NP domain targeted	NP amino acid	vRNP activity
NPrbp1 – RNA binding pocket	R152A	YES
NPbd1 – body domain	E46A, K48A	YES
NPbd2 – body domain	D101A	YES
NPbd3 – body domain	Y289G, K293G, E294G, Q308G, N309G	NO
NPbd4 – body domain	S482A	YES
NPbd5 – body domain	S467G, E469G, N470G, T471G, N472G	YES
NPhd1 – head domain	R213A, K214A	YES

We next examined the activity of reconstituted vRNPs comprised of NPbd3 at the level of RNA. We confirm the defect in viral protein expression observed with NPbd3 is reflected at the RNA level. To directly examine both viral plus sense RNA synthesis (c/mRNA) and the second step in replication, negative sense RNA synthesis (vRNA), the reconstituted expression system was carried out with either an influenza M cRNA or M vRNA template. RNA was isolated from cells, DNAse treated, reverse transcribed with oligo dT (vRNA template) or vRNA specific primer (cRNA template), and subjected to qPCR analysis targeting the M gene. NPbd3 was found to be defective for viral c/mRNA synthesis and vRNA synthesis compared to WT NP (Fig. 3b and c).

### Characterization of NP body domain mutant #3

#### Conservation

To analyze the peptide sequence conservation of this domain, we utilized analysis tools from the Influenza Research Database, a resource containing “all genomic and proteomic data available in public repositories for influenza viruses” [40]. We first ran analysis for sequence variation and confirmed that these 5 amino acids are highly conserved in human influenza viruses. Position 289 is the least conserved with Tyrosine encoded in many influenza strains but Histidine in others. While Tyr and His are characterized with different properties (polar versus charged, respectively) both contain an aromatic ring structure. When examining variation at 289 in human influenza subtypes, H1N1 and pandemic H1N1 preferentially encode His while H1N2, H2N2, H3N2, H5N1, and H7N9 preferentially encode Tyr. There are only 5 sequences in the database that are unknown at this position (Xaa), with two others encoding Lysine (both from pH1N1), while 15,057 encode His and 11,096 encode Tyr. Position 293 is charged conserved, with strains encoding either Arginine or Lysine. H1N1, pH1N1, and H5N1 preferentially encode Arg while H1N1, H2N2, H3N2 preferentially encode Lysine. Only 4 sequences in the database do not encode Arg or Lys at this position (unknown Xaa), while 15,733 encode Arg and 9824 encode Lys. Position 294 is highly conserved, with all subtypes preferentially encoding Glutamic acid at this position. Only 1 sequence was found to encode Valine instead (from pH1N1), with 5 sequences reported as unknown, and 26,154 encoding Glu. Position 308 is Glutamate in all subtypes, with just 8 sequences reported as unknown and 26,153 encoding Gln. Position 309 is preferentially Asparagine in all subtypes, but does have some variation, especially among H1N1 sequences. H1N1 subtypes encode Asparagine in most sequences reported (7751) but also Ala (5),His (1),Ile (2),Ser (2), and Thr (1559). Both H3N2 and pH1N1 also have variant sequences reported, with Thr (2) and His (1). Overall this position is conserved, with 24,585 encoding Asn and 1572 encoding Thr, an amino acid with similar properties to Asn, and only 17 encoding other amino acids or unknown. We further ran short peptide searches against the pre-compiled IRD database for influenza A NP segments. We found 28,365 matches to amino acids 289–294 when searching with the sequence Yxxx[RK]E. and 39,909 matches with [YH]xxx[RK]E. For amino acids 307–310 we found 38,085 exact matches to the sequence LQNS. Our protein sequence analysis confirms this region of NP is highly conserved.

#### Expression and localization

**Fig. 4:**
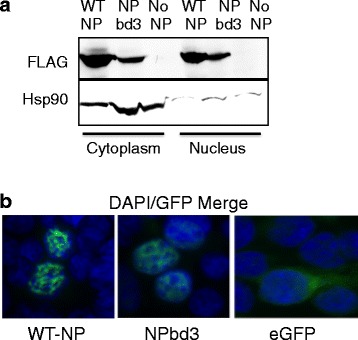
NPbd3 is expressed and localized as wild type NP. **a** Cells expressing reconstituted vRNPs were collected and fractionated with NP-40 non-ionic detergent to break open cellular plasma membrane. Microscopy was used to confirm disrupted plasma membranes and intact nuclei. Nuclei were pelleted by centrifugation and proteins isolated. Proteins were separated on a 10% SDS PAGE gel and transferred to nitrocellulose. Western was performed with anti-FLAG to detect WT NP and NPbd3 and anti-Hsp90 to detect Hsp90, a protein localized in the cytoplasm, which serves as confirmation of cellular fractionation. Expected size was confirmed by comparison with Fisher BioReagents EZ-Run protein standards. **b** NP-GFP and NPbd3-GFP fusion proteins or eGFP as indicated were expressed in cells grown on poly-L-Lysine cover slips. Cells were washed and fixed using a 1:1 methanol and acetone mixture. The coverslips were mounted onto glass slides using SouthernBiotech™ Dapi-Fluoromount-G™ Clear Mounting Media which stains the cell nucleus blue. Slides were observed on a Nikon ECLIPSE TE2000-U fluorescent microscope and images were captured with an Andor Clara DR-3446 camera using NIS-Elements AR software

To ensure NPbd3 was expressed and localized as wild type NP (WT-NP) we performed cellular fractionation and Western blot. Cells were collected and fractionated using non-ionic detergent and centrifugation as in materials and methods. Cellular fractions were analyzed by Western blot using anti-FLAG to observe NP and anti-Hsp90 to demonstrate protein localized to the cytoplasm. NPbd3 is expressed and localized in the nucleus as WT-NP (Fig. 4a). To further confirm localization of NPbd3, NP-GFP and NPbd3-GFP fusion proteins were constructed and observed through fluorescence microscopy. Again NPbd3 was localized in a pattern similar to WT-NP 48 h post transfection (Fig. 4b).

**Fig. 5:**
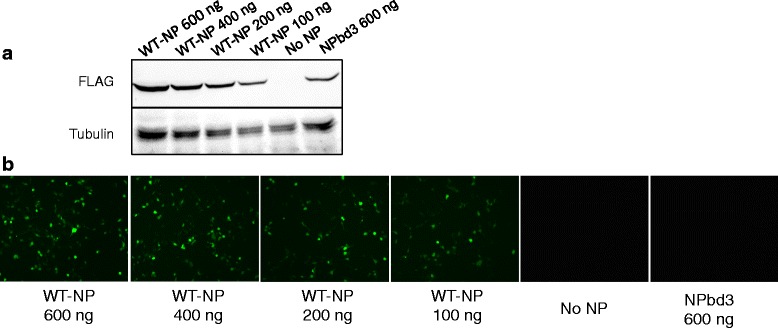
Titration of NP and reconstituted vRNP activity. 293 T cells were transfected with plasmids to express reconstituted vRNPs with GFP-M vRNA and either WT-NP, no NP, or NPbd3 at concentrations indicated. **a** Total protein from titration samples was isolated at 48 h post transfection and run on a 10% SDS PAGE and transferred to nitrocellulose. The membrane was probed with FLAG to identify NP and Tubulin as the loading control. Expected size was confirmed by comparison with Fisher BioReagents EZ-Run protein standards. **b** Cells were observed for GFP-M expression 48 h post transfection. WT-NP expressed by transfection of 400 ng plasmid is the standard concentration used in our reconstituted vRNP experiments and serves as positive control, while no NP is the negative control. GFP was visualized with a Nikon Eclipse TS100 (Nikon Intensilight C-HGFI for fluorescence) inverted microscope and images captured with the Nikon DS-Qi1Mc camera with NS Elements software

NPbd3 was expressed at a slightly lower level than WT-NP in many of our location experiments (Fig. 4). To verify that NP is expressed in excess in the reconstituted vRNP assay and confirm decreased expression of NPbd3 was not responsible for the defect observed, we titrated WT-NP expressing plasmid in transfection from 600 nanograms to 100 nanograms. As expected, lowering plasmid amount during transfection did result in decreased NP protein expression (Fig. 5a). We examined activity in the reconstituted expression system using a GFP MvRNA template and confirm that even transfection of 100 ng DNA plasmid resulted in little change in GFP expression (Fig. 5b). While 100 ng of NP expression plasmid and 600 ng NPbd3 expression plasmid result in similar NP protein expression (Fig. 5a), the two samples do not have similar vRNP activity as represented by GFP expression (Fig. 5b). This experiments confirms NP is in excess in our standard reconstituted vRNP assay and that the lower NPbd3 expression is not the cause of the lack of vRNP activity observed.

#### Nucleic acid binding and oligomerization

**Fig. 6:**
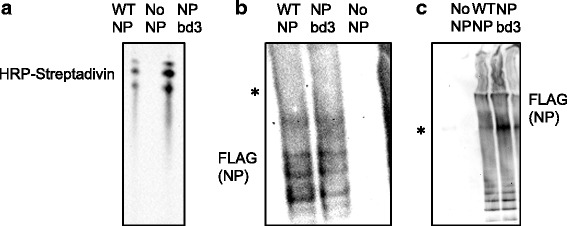
NPbd3 maintains nucleic acid binding, oligmerization, and vRNP formation. **a** WT NP and NPbd3 were immuno purified using anti-FLAG antibody. Purified WT NP and NPbd3 were incubated with biotin labeled single stranded DNA and separated on an 8% PAGE TBE non denaturing gel before transfer to nitrocellulose. Biotin ssDNA was detected by interaction with Streptavidin-HRP and ECL reagents. No NP samples expressed no NP protein and serve as negative control. **b** Blue Native Gel Electrophoresis was utilized to migrate protein extracts of cells expressing NP and NPbd3 followed by western blot with anti-FLAG antibody to demonstrate NP oligomer formation. Asterisk represents Native Mark Unstained Protein standard at 480 kDa. **c** Blue Native Gel Electrophoresis was utilized to migrate protein extracts of cells expressing reconstituted vRNPs comprised of either NP or NPbd3 followed by western blot with anti-FLAG antibody to demonstrate vRNP formation. Asterisk represents Native Mark Unstained Protein standard at 1048 kDa

NP binds viral RNA via the nucleic acid phosphate back bone and through NP-NP oligomerization provides the basic structure for the vRNP. There is one NP monomer for approximately 24 nucleotides of RNA [32]. To confirm NPbd3 did not result in a defect in RNA binding, we performed an electrophoretic mobility shift assay (EMSA) with single stranded DNA corresponding to the 5’ and 3’ ends of the viral RNA. NP RNA binding is blocked by saturation with single stranded DNA, demonstrating both nucleic acids bind at the same nucleic acid binding pocket [23]. WT-NP and NPbd3 were immunopurified with FLAG antibody coupled to agarose beads and eluted with FLAG peptide. Immunopurified NP proteins were analyzed by SDS-PAGE and Coomassie Blue stain to ensure no additional protein contaminants (data not shown). Incubation of purified proteins with biotin labeled single stranded nucleic acid followed by non-denaturing native PAGE and western blot with HRP- Streptavidin confirmed NPbd3 bound nucleic acids as WT-NP (Fig. 6a). Biotin ssDNA with no bound protein was consistently not detected in our assay; it is probable unbound ssDNA does not transfer and stay attached to nitrocellulose membranes as well as that associated with protein under our assay and detection conditions. Although NPbd3 appears to shift more RNA than WT-NP, this is likely a reflection of protein input and was inconsistent in repeated trials. Furthermore, these assays do not assess the kinetics of RNA binding only that NPbd3 maintains nucleic acid binding as expected, given we did not alter the known RNA binding domain. NP oligomerization is important for RNA binding and formation of functional vRNPs. To examine NP oligomer formation, protein extracts from cells expressing NP or NPbd3 were separated by blue native polyacrylamide gel electrophoresis (BN-PAGE) followed by western blot with anti-FLAG antibody. Results demonstrate the ability of WT-NP and NPbd3 to form oligomers (Fig. 6b). We also used blue native gel electrophoresis to confirm high molecular weight vRNP complexes are formed. Total protein extracts from cells expressing vRNPs comprised of the viral RNA dependent RNA polymerase (PA, PB1, PB2) along with M vRNA, and either WT-NP, no NP, or NPbd3 were separated by blue native polyacrylamide gel electrophoresis (BN-PAGE) followed by western blot with anti-FLAG antibody to detect NP containing complexes. NPbd3 is capable of formation of high molecular weight vRNP complexes similar to wild type NP (Fig. 6c), as expected given NPbd3 binds nucleic acids (Fig. 6a) and oligomerizes (Fig. 6b).

Although NPbd3 was expressed in the cell, localized as WT-NP, found in oligomers, and bound nucleic acid *in vitro*, NPbd3 was completely defective for viral RNA expression in reconstituted vRNPs and cRNPs. We next decided to further define the important residues within this domain of NP.

### NP body domain single mutant analysis

**Fig. 7:**
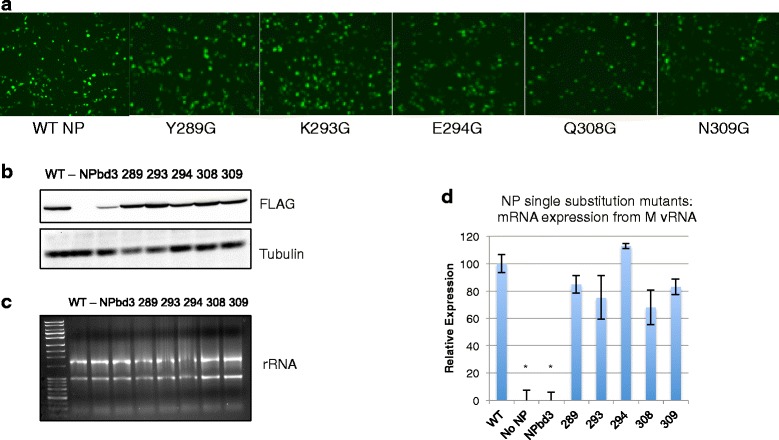
NP body domain single amino acid substitution mutants display near wild type activity in reconstituted vRNP assay. **a** Cells expressing reconstituted vRNPs with GFP-M vRNA were visualized for GFP expression with a Nikon Eclipse TS100 (Nikon Intensilight C-HGFI for fluorescence) inverted microscope and images captured with the Nikon DS-Qi1Mc camera with NS Elements software. Number indicates NP single residue mutated. **b** Total protein was isolated from cells expressing reconstituted vRNPs with GFP-M vRNA template and the indicated NP substitution mutant. Total protein extract was separated on 10% SDS PAGE and transferred to nitrocellulose. WT NP, NPbd3, and NP single mutants were detected with anti-FLAG. Anti-tubulin was used as loading control. Expected size was confirmed by comparison with Fisher BioReagents EZ-Run protein standards. **c** RNA from cells expressing reconstituted vRNPs with FLAG-M vRNA and either WT NP, no NP, or NPbd3, or the single amino acid substitution mutants were isolated and analyzed by 1% bleach/agarose gel electrophoresis. **d** One microgram of RNA was treated with DNase before being reverse transcribed with oligo dT (mRNA) and analyzed through quantitative PCR with primers targeting the M gene. qPCR reactions were carried out in triplicate. Significance was evaluated through t-test by comparing WT NP; asterisks indicate *p*-values <0.01

To investigate this region of the NP body domain further, single amino acid glycine substitution mutants were cloned, Y289G, K293G, E294G, Q308G, and N309G. These single mutants were analyzed for vRNP function by assessing GFP expression using our reconstituted vRNP expression system with GFP-M vRNA (Fig. 2). All five single NP mutants displayed activity similar to WT-NP (Fig. 7a). Protein analysis showed the NP single mutants were expressed at levels similar to WT-NP (Fig. 7b). RNA concentration and integrity was confirmed with gel electrophoresis (Fig. 7c). RNA analysis of the M gene through reverse transcription using oligo-dT primers and qPCR revealed levels of viral mRNA expression with no statistical difference compared to WT-NP (Fig. 7d). Therefore, our analysis of NP single substitutions within this region of the body domain revealed that all were nearly as functional as WT-NP, suggesting these accessible amino acids play a redundant role.

### NP body domain double mutant analysis

**Fig. 8:**
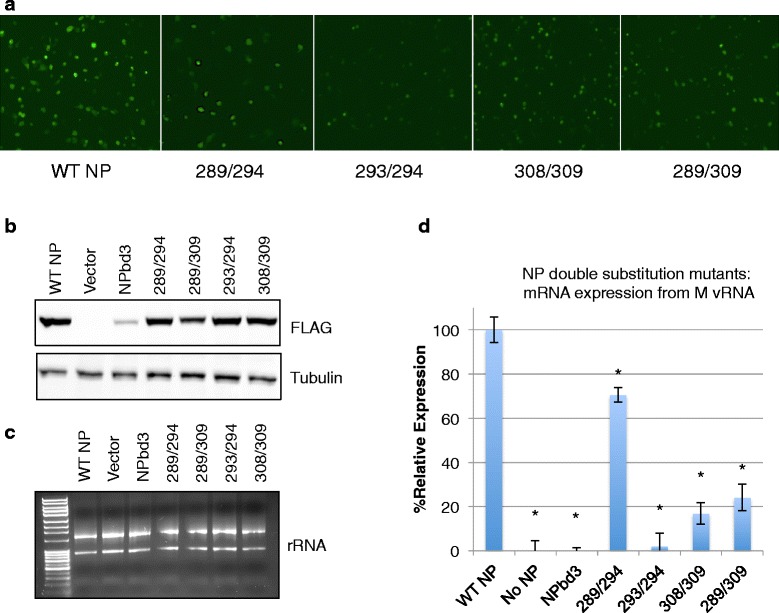
NP double amino acid substitution mutants display decreased activity in reconstituted vRNP assay. **a** Cells expressing reconstituted vRNPs with GFP-M vRNA were visualized for GFP expression with a Nikon Eclipse TS100 (Nikon Intensilight C-HGFI for fluorescence) inverted microscope and images captured with the Nikon DS-Qi1Mc camera with NS Elements software. Number indicates the two NP residues substituted with glycine. **b** Total protein was isolated from cells expressing reconstituted vRNPs with GFP-M vRNA template and the indicated NP mutant. Total protein extract was separated on 10% SDS PAGE and transferred to nitrocellulose. WT NP, NPbd3, and NP double mutants were detected with anti-FLAG. Anti-tubulin was used as loading control. Expected size was confirmed by comparison with Fisher BioReagents EZ-Run protein standards. **c** RNA from cells expressing reconstituted vRNPs with FLAG-M vRNA and either WT NP, no NP, or NPbd3, or the double amino acid mutants were isolated and analyzed by 1% bleach/agarose gel electrophoresis. **d** One microgram of RNA was treated with DNase before being reverse transcribed with oligo dT (mRNA) and analyzed through quantitative PCR with primers targeting the M gene. qPCR reactions were carried out in triplicate. Significance was evaluated through t-test by comparing WT NP; asterisks indicate *p*-values <0.02

We next altered pairs of amino acids within this region of the NP body domain to further define which residues contribute to the defect observed. Four body domain double mutants were constructed, Q308G/N309G, Y289G/E294G, Y289G/N309G, and K293G/E294G. These double mutants were analyzed for vRNP function by assessing GFP expression using our reconstituted vRNP expression system with GFP-M vRNA (Fig. 2). All NP double mutants showed statistically less GFP expression than WT-NP (Fig. 8a). Total protein analysis by western blot revealed that all NP double mutants are expressed as WT-NP (Fig. 8b). RNA concentration and integrity was confirmed with gel electrophoresis (Fig. 8c). RNA analysis of the M gene through reverse transcription using oligo-dT primers and qPCR revealed a significant decrease in viral c/mRNA expression in all double mutants compared to WT-NP (Fig. 8d). Analysis of NP double mutants supports the defect in viral RNA expression observed with NPbd3 is likely caused by a combination of multiple accessible amino acids in the NP body domain.

## Discussion

Substitution analysis of the NP body domain revealed a region of five amino acids that is essential for viral gene expression. NPbd3 encodes glycine at 5 amino acids within an accessible region of the NP body domain. Cellular fractionation and western blot, in addition to NP-GFP fusions and fluorescence, confirm NPbd3 was expressed and localized in the nucleus as WT-NP (Fig. 4). EMSA with purified NP protein confirm NPbd3 bound nucleic acids as WT-NP (Fig. 6a). Blue Native Gel Electrophoresis demonstrated NPbd3 could oligomerize and form vRNPs (Fig. 6b and c). Although NPbd3 was expressed, localized, and bound nucleic acid as WT-NP, we found NPbd3 was completely defective for RNA expression in reconstituted vRNPs and cRNPs as evaluated by reverse transcription and quantitative polymerase chain reaction (RT-qPCR).

To investigate this region of the NP body domain further, single and double amino acid mutations were cloned. Analysis of NP single mutants revealed that all were nearly as functional as WT-NP for RNA expression in reconstituted vRNPs, suggesting these accessible amino acids in the NP body domain play a redundant role (Fig. 7). However, four different combinations of two amino acid mutations resulted in NP double mutants that displayed a significant defect in RNA expression in reconstituted vRNPs, confirming these accessible amino acids in the NP body domain play a significant role for viral RNA synthesis (Fig. 8). The greatest defect in mRNA synthesis observed was in double mutant 293/294, followed by 308/309, 289/309, with 289/294 demonstrating minimal defect.

The body domain region designated for mutagenesis in NPbd3 was selected for probable interaction with the RdRP based on mini-replicon images [37]. Residues K293 and E294 are charge conserved. A change to glycine at either residue was not enough to inhibit vRNP function, and therefore these residues may participate in intermolecular ionic interactions. The tyrosine at residue 289, glutamine at residue 308, and asparagine at residue 309 are polar: substituting a glycine at two of these residues leads to a defect in mRNA synthesis. We hypothesize that the three amino acid residues (Y289G, Q308G, and N309G) are redundant for NP interaction or function with the viral RdRP, specifically PB2, and disruption of this essential NP interaction is the explanation for the RNA defect observed with mutations at 308/309 and 289/309. Evidence to support this includes experiments in which passaged avian influenza revealed that NP N309K change is consistently paired with specific changes in PB2 (PB2 Q236H, E627K), which result in avian influenza replicating more efficiently in human host cells [36]. Future experiments will focus on obtaining direct interaction data through co-immunoprecipitation of our double NP substitution mutants with PB2 to biochemically confirm and better define this interaction. Our data suggest we have identified two different interaction surfaces on the body domain of NP. One comprised of the ionic interaction potential with residues K293 and E294 and the other comprised of polar interaction potential with residues Y289, Q308, and N309. This is supported by our result with NP double substitution mutant Y289G/E294G, which demonstrates minimal defect in vRNP activity. This is consistent if indeed each substitution is part of a unique interaction domain comprised of additional redundant amino acids as our data indicate.

We reason that the identified residues within the accessible body domain of NP comprise important dynamic NP interaction surfaces and small molecule inhibitors could block access to these NP interactions, making this region a viable antiviral target. Indeed, two amino acids within the polar interaction site of this NP body domain, 289 and 309, comprise a novel groove implicated in binding the small molecule inhibitor nucleozin [33]. The influenza A(H1N1)pdm09 strains contained a rare polymorphism at residue 289 [33]. Instead of a tyrosine, these H1N1 strains contain a histidine at amino acid 289. Tyrosine has a large hydrophobic ring structure side chain with a polar OH group while histidine has a smaller and positively charged side chain. This change likely disrupts the aromatic ring stacking between nucleozin and the tyrosine at 289 [33]. Although NP N309K resulted in rescue of inhibition from nucleozin, our data reveal this genetic change is possible because N309 is part of a polar interaction site with redundant amino acids for vRNP function. However, our results confirm this novel groove is essential for viral gene expression and should be considered as a viable antiviral target with improved inhibitors. Beyond nucleozin, another compound, FA-10 showed efficacy against the A(H1N1)pdm09 strains but less activity toward wild-type WSN virus [33].

## Conclusions

Our data demonstrate that optimization of compounds targeting this domain could lead to a novel and effective antiviral therapy to combat emerging current antiviral resistant influenza subtypes. Residues within this NP domain are redundant for vRNP function and therefore small molecule inhibitors cannot rely on only one residue for interaction with this pocket, or escape mutants will be likely. Instead, an inhibitor that takes advantage of the multiple redundant interaction potentials within this domain would make escape mutants less likely. The work presented here has defined this domain with clarity, providing evidence that the polar amino acids 289, 308, and 309 offer one target while the charged interaction at 293 and 294 represents a separate target; both regions of the domain can be pursued in a combinational therapeutic approach. This project demonstrates the importance of this NP accessible domain for viral gene expression, validating studies to identify inhibitors of this domain to block influenza virus. The work here supports novel antivirals targeting this conserved NP body domain are a viable approach to counter emerging influenza viruses as existing antivirals prove no longer efficacious.

## Abbreviations

BN-PAGE: Blue native polyacrylamide gel electrophoresis
EMSA: Electrophoretic mobility shift assay
GFP: Green Fluorescent Protein
NP: Nucleoprotein
RdRP: RNA dependent RNA polymerase
RT-qPCR: Reverse transcription quantitative polymerase chain reaction
vRNP: Viral ribonucleoprotein

## References

[1] Centers for Disease Control and Prevention. http://www.cdc.gov/flu/avianflu/avian-in-humans.htm. Accessed 15 June 2016.

[2] Centers for Disease Control and Prevention. http://www.cdc.gov/flu/about/viruses/transmission.htm. Accessed 15 June 2016.

[3] Neumann G, Noda T, Kawaoka Y (2009). Emergence and pandemic potential of swine-origin H1N1 influenza virus. Nature.

[4] Centers for Disease Control and Prevention. http://www.cdc.gov/flu/professionals/vaccination/effectiveness-studies.htm. Accessed 15 June 2016.

[5] Centers for Disease Control and Prevention. http://www.cdc.gov/media/releases/2016/s0622-laiv-flu.html. Accessed 15 July 2016.

[6] Hunter JC, Rodríguez DC, Aragón TJ. Public health management of antiviral drugs during the 2009 H1N1 influenza pandemic: a survey of local health departments in California. BMC Public Health. 2012;12(82). doi: 10.1186/1471-2458-12-82. 10.1186/1471-2458-12-82 PMC3323435 22276659

[7] Centers for Disease Control and Prevention. http://www.cdc.gov/flu/pastseasons/1314season.htm. Accessed 15 June 2016.

[8] Pizzorno A, Abed Y, Bouhy X, Beaulieu É, Mallett C, Russell R, Boivin G (2012). Impact of mutations at residue I223 of the neuraminidase protein on the resistance profile, replication level, and virulence of the 2009 pandemic influenza virus. Antimicrob Agents Chemother.

[9] van der Vries E, Kroeze EJV, Stittelaar KJ, Linster M, Van der Linden A, Schrauwen EJ, Leijten LM, van Amoerogen G, Schutten M, Kuiken T, Osterhaus AD, Fouchier RA, Boucher CA, Herfst S (2011). Multidrug resistant 2009 A/H1N1 influenza clinical isolate with a neuraminidase I223R mutation retains its virulence and transmissibility in ferrets. PLoS Pathog.

[10] Baz M, Abed Y, Papenburg J, Bouhy X, Hamelin MÈ, Boivin G (2009). Emergence of oseltamivir-resistant pandemic H1N1 virus during prophylaxis. N Engl J Med.

[11] Kiso M, Mitamura K, Sakai-Tagawa Y, Shiraishi K, Kawakami C, Kimura K, Hayden FG, Suguya N, Kawaoka Y (2004). Resistant influenza A viruses in children treated with oseltamivir: descriptive study. Lancet.

[12] Davis AM, Chabolla BJ, Newcomb LL. Emerging antiviral resistant strains of influenza A and the potential therapeutic targets within the viral ribonucleoprotein (vRNP) complex. Virol J. 2014;1(167). doi:10.1186/1743-422X-11-167. 10.1186/1743-422X-11-167 PMC4180549 25228366

[13] Dubois J, Terrier O, Rosa-Calatrava M (2014). Influenza viruses and mRNA splicing: doing more with less. MBio.

[14] Chen W, Calvo PA, Malide D, Gibbs J, Schubert U, Bacik I, Basta S, O’Neill R, Schickli J, Palese P, Henklein P, Bennink JR, Yewdell JW (2001). A novel influenza A virus mitochondrial protein that induces cell death. Nat Med.

[15] Wise HM, Foeglein A, Sun J, Dalton RM, Patel S, Howard W, Anderson EC, Barclay WS, Digard P (2009). A complicated message: identification of a novel PB1-related protein translated from influenza A virus segment 2 mRNA. J Virol.

[16] Jagger BW, Wise HM, Kash JC, Walters KA, Wills NM, Xiao YL, Dunfee RL, Schartzman LM, Ozinsky A, Bell GL, Dalton RM, Lo A, Efstanthiou S, Atkins JF, Firth AE, Taubenberger JK, Digard P (2012). An overlapping protein-coding region in influenza A virus segment 3 modulates the host response. Science.

[17] Muramoto Y, Noda T, Kawakami E, Akkina R, Kawaoka Y (2012). Identification of novel influenza A virus proteins translated from PA mRNA. J Virol.

[18] Li ML, Rao P, Krug RM (2001). The active sites of the influenza cap-dependent endonuclease are on different polymerase subunits. EMBO J.

[19] Plotch SJ, Bouloy M, Ulmanen I, Krug RM (1981). A unique cap (m7GpppXm)-dependent influenza virion endonuclease cleaves capped RNAs to generate the primers that initiate viral RNA transcription. Cell.

[20] Yuan P, Bartlam M, Lou Z, Chen S, Zhou J, He X, Lv Z, Ge R, Li X, Deng T, Fodor E, Rao Z, Liu Y (2009). Crystal structure of an avian influenza polymerase PA_N_ reveals an endonuclease active site. Nature.

[21] Dias A, Bouvier D, Crépin T, McCarthy AA, Hart DJ, Baudin F, Cusack S, Ruigrok RW (2009). The cap-snatching endonuclease of influenza virus polymerase resides in the PA subunit. Nature.

[22] Poon LL, Pritlove DC, Fodor E, Brownlee GG (1999). Direct evidence that the poly (A) tail of influenza A virus mRNA is synthesized by reiterative copying of a U track in the virion RNA template. J Virol.

[23] Newcomb LL, Kuo RL, Ye Q, Jiang Y, Tao YJ, Krug RM (2009). Interaction of the influenza a virus nucleocapsid protein with the viral RNA polymerase potentiates unprimed viral RNA replication. J Virol.

[24] Robb NC, Chase G, Bier K, Vreede FT, Shaw PC, Naffakh N, Schwemmle M, Fodor E (2011). The influenza A virus NS1 protein interacts with the nucleoprotein of viral ribonucleoprotein complexes. J Virol.

[25] Naito T, Kiyasu Y, Sugiyama K, Kimura A, Nakano R, Matsukage A, Nagata K (2007). An influenza replicon system in yeast identified Tat-SF1 as a stimulatory host factor for viral RNA synthesis. Proc Natl Acad Sci.

[26] Wang P, Song W, Mok BW, Zhao P, Qin L, Lai A, Smith GK, Zhang J, Lin T, Guan Y, Chen H (2009). Nuclear factor 90 negatively regulates influenza virus replication by interaction with the viral nucleoprotein. J Virol.

[27] Momose F, Basler CF, O’Neill RE, Iwamatsu A, Palese P, Nagata K (2001). Cellular splicing factor RAF-2p48/NPI-5/BAT1/UAP56 interacts with the influenza virus nucleoprotein and enhances viral RNA synthesis. J Virol.

[28] Kawaguchi A, Momose F, Nagata K (2011). Replication-coupled and host factor-mediated encapsidation of the influenza virus genome by viral nucleoprotein. J Virol.

[29] Wisskirchen C, Ludersdorfer TH, Müller DA, Moritz E, Pavlovic J (2011). The cellular RNA helicase UAP56 is required for prevention of double-stranded RNA formation during influenza A virus infection. J Virol.

[30] Kukol A, Hughes DJ (2014). Large-scale analysis of influenza A virus nucleoprotein sequence conservation reveals potential drug-target sites. Virology.

[31] Ye Q, Guu TS, Mata DA, Kuo RL, Smith B, Krug RM, Tao YJ (2013). Biochemical and structural evidence in support of a coherent model for the formation of the double-helical influenza A virus ribonucleoprotein. MBio.

[32] Ye Q, Krug RM, Tao YJ (2006). The mechanism by which influenza A virus nucleoprotein forms oligomers and binds RNA. Nature.

[33] Kao RY, Yang D, Lau LS, Tsui WH, Hu L, Dai J, Chan MP, Chan CM, Wang P, Zheng BJ, Sun J, Huang JD, Madar J, Chen G, Chen H, Guan Y, Yuen KY (2010). Identification of influenza A nucleoprotein as an antiviral target. Nat Biotechnol.

[34] Shen YF, Chen YH, Chu SY, Lin MI, Hsu HT, Wu PY, Wu CJ, Liu HW, Lin FY, Lin G, Hsu PH, Yang AS, Cheng YS, Wu YT, Wong CH, Tsai MD (2011). E339… R416 salt bridge of nucleoprotein as a feasible target for influenza virus inhibitors. PNAS.

[35] Biswas SK, Boutz PL, Nayak DP (1998). Influenza virus nucleoprotein interacts with influenza virus polymerase proteins. J Virol.

[36] Danzy S, Studdard LR, Manicassamy B, Solorzano A, Marshall N, García-Sastre A, Steel J, Lowen AC (2014). Mutations to PB2 and NP proteins of an avian influenza virus combine to confer efficient growth in primary human respiratory cells. J Virol.

[37] Coloma R, Valpuesta JM, Arranz R, Carrascosa JL, Ortín J, Martín-Benito J (2009). The structure of a biologically active influenza virus ribonucleoprotein complex. PLoS Pathog.

[38] Sanchez A, Guerrero-Juarez CF, Ramirez,J, Newcomb LL. Nuclear localized Influenza nucleoprotein N-terminal deletion mutant is deficient in functional vRNP formation. Virol J. 2014;11(1). doi:10.1186/1743-422X-11-155. 10.1186/1743-422X-11-155 PMC4177073 25174360

[39] Aranda PS, LaJoie DM, Jorcyk CL (2012). Bleach gel: a simple agarose gel for analyzing RNA quality. Electrophoresis.

[40] Influenza Research Database. www.fludb.org. Accessed 15 Jan 2017.

